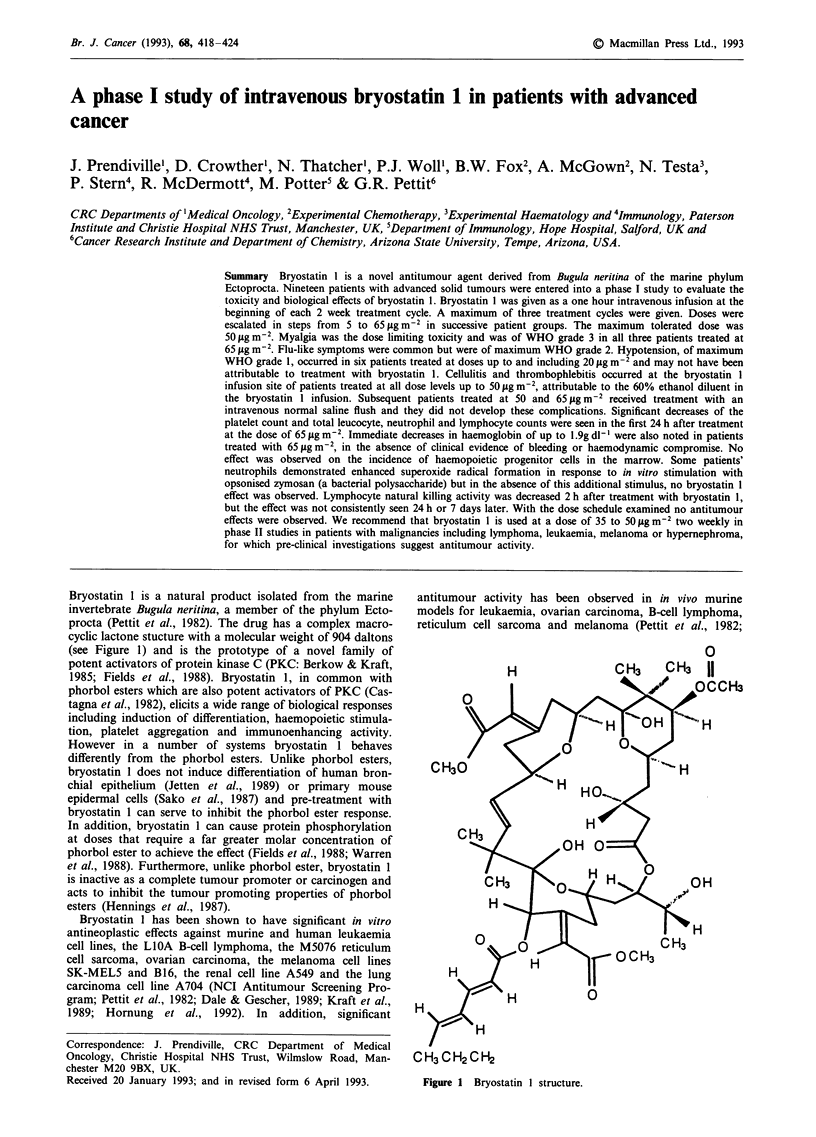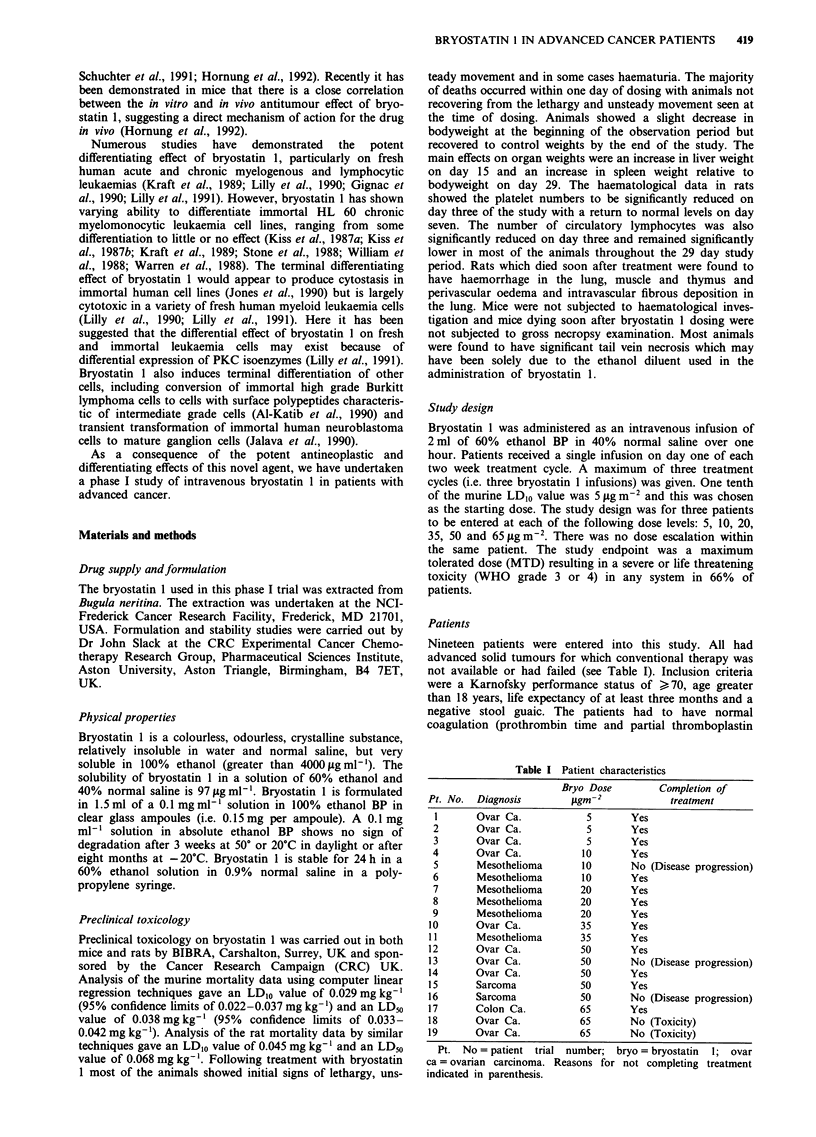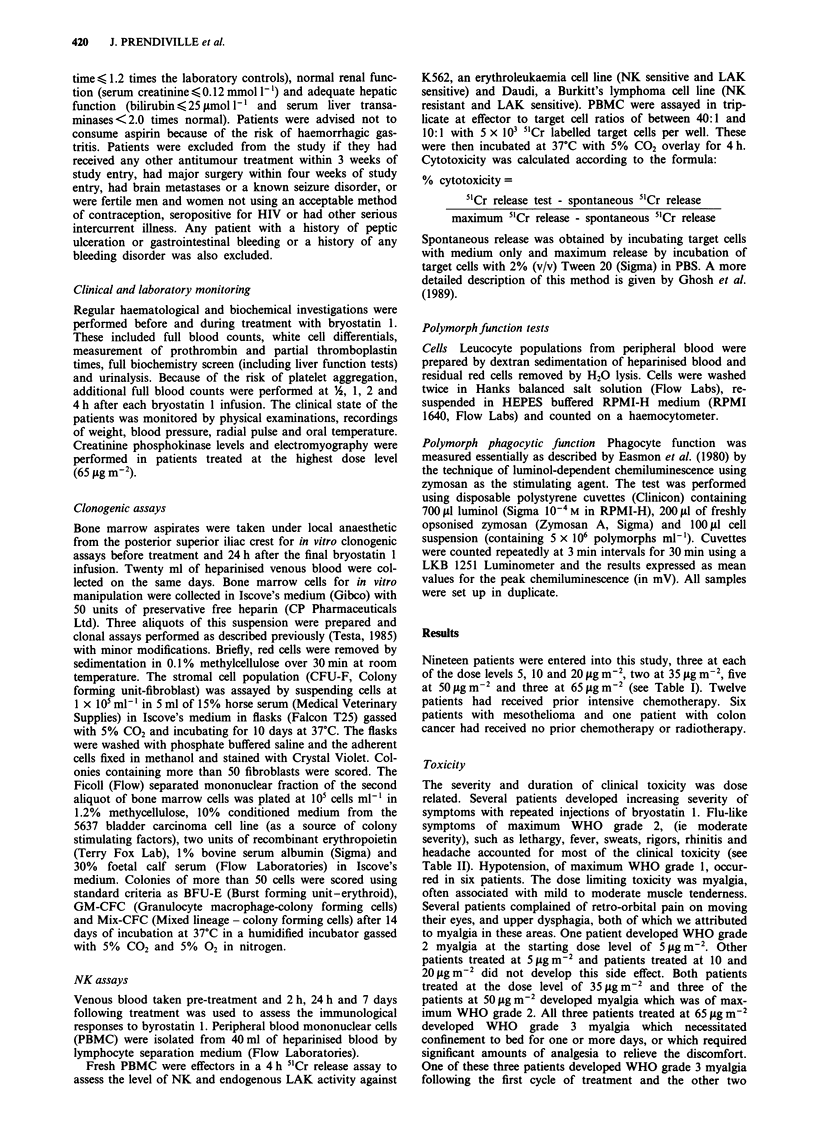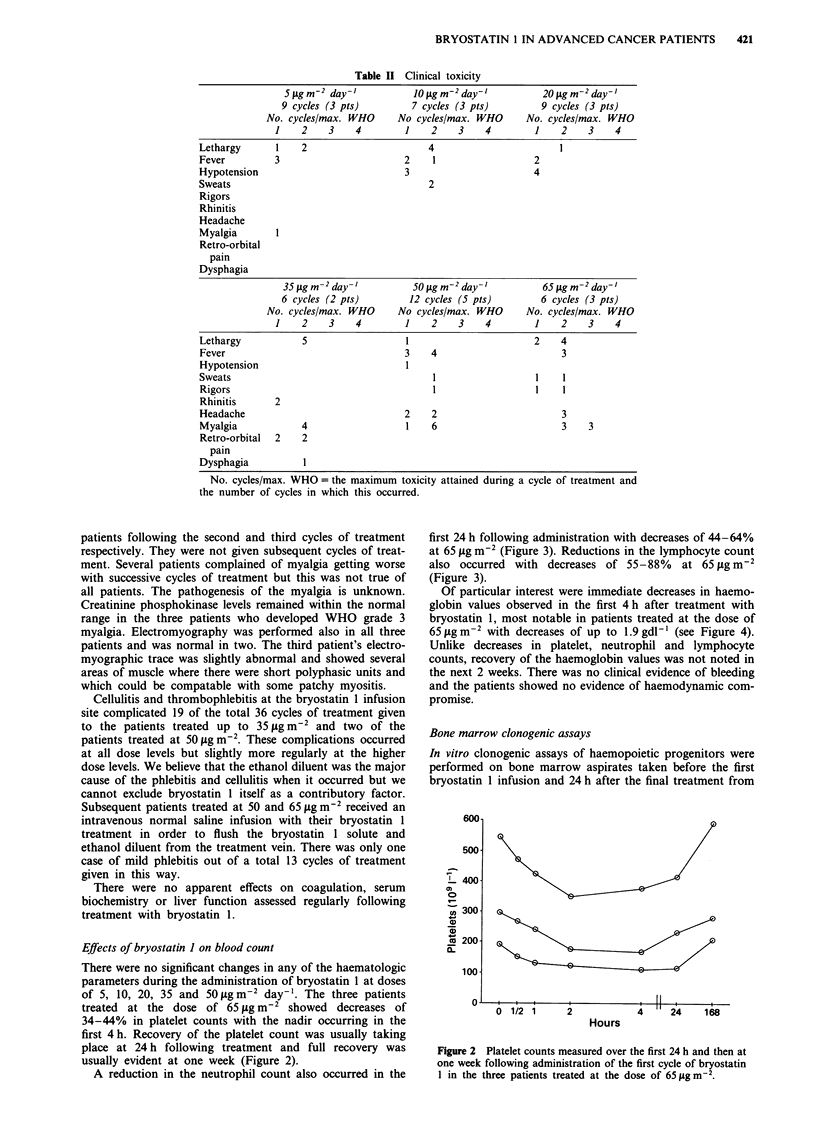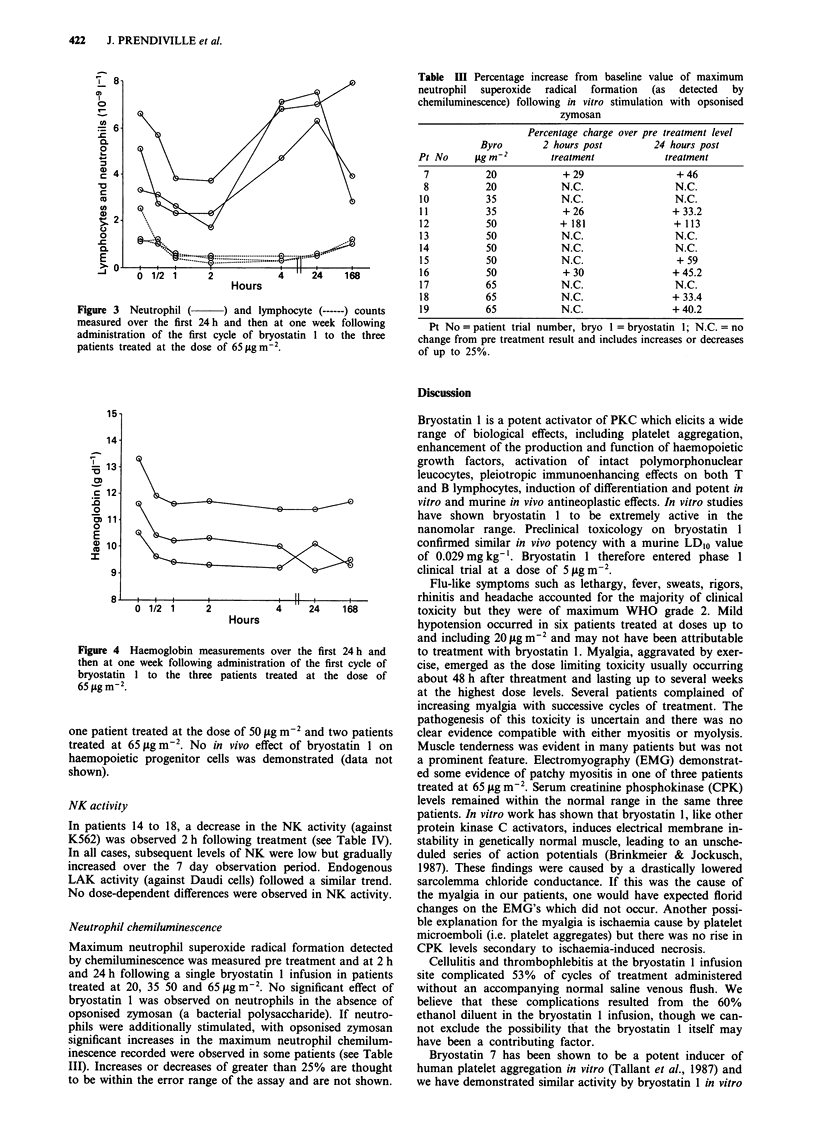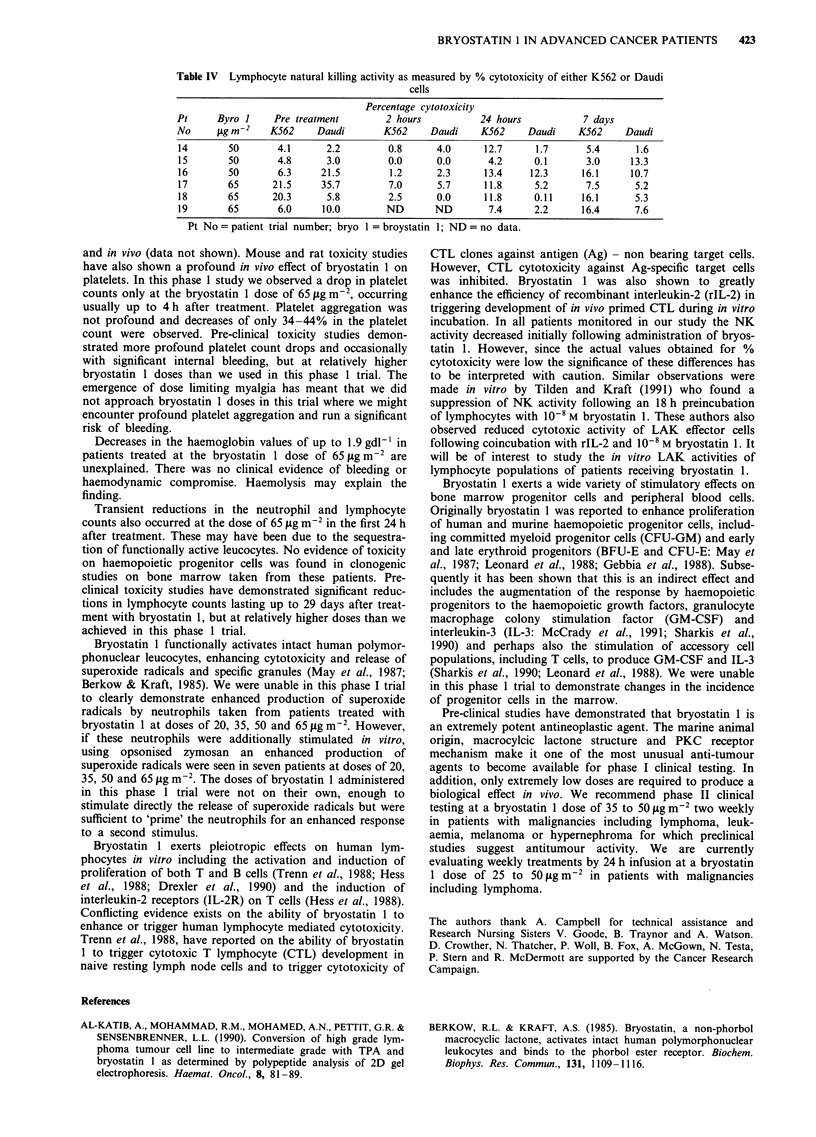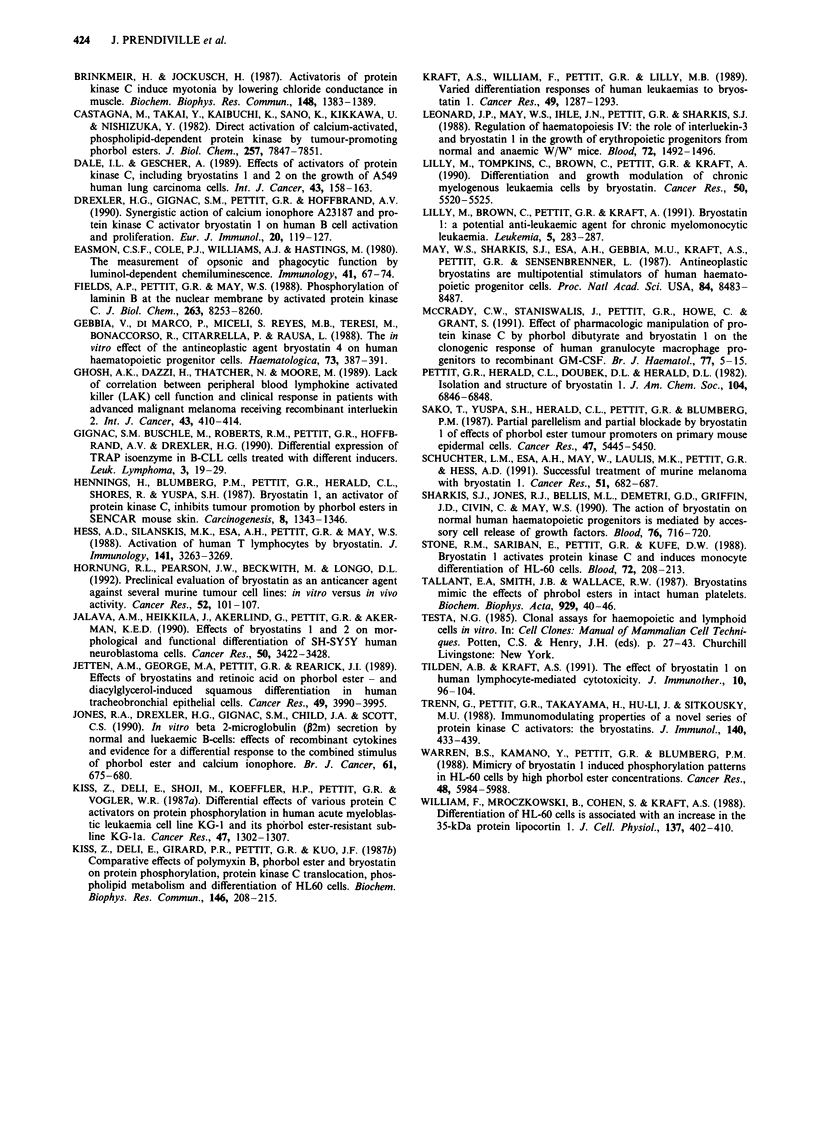# A phase I study of intravenous bryostatin 1 in patients with advanced cancer.

**DOI:** 10.1038/bjc.1993.352

**Published:** 1993-08

**Authors:** J. Prendiville, D. Crowther, N. Thatcher, P. J. Woll, B. W. Fox, A. McGown, N. Testa, P. Stern, R. McDermott, M. Potter

**Affiliations:** CRC Department of Medical Oncology, Paterson Institute and Christie Hospital NHS Trust, Manchester, UK.

## Abstract

Bryostatin 1 is a novel antitumour agent derived from Bugula neritina of the marine phylum Ectoprocta. Nineteen patients with advanced solid tumours were entered into a phase I study to evaluate the toxicity and biological effects of bryostatin 1. Bryostatin 1 was given as a one hour intravenous infusion at the beginning of each 2 week treatment cycle. A maximum of three treatment cycles were given. Doses were escalated in steps from 5 to 65 micrograms m-2 in successive patient groups. The maximum tolerated dose was 50 micrograms m-2. Myalgia was the dose limiting toxicity and was of WHO grade 3 in all three patients treated at 65 micrograms m-2. Flu-like symptoms were common but were of maximum WHO grade 2. Hypotension, of maximum WHO grade 1, occurred in six patients treated at doses up to and including 20 micrograms m-2 and may not have been attributable to treatment with bryostatin 1. Cellulitis and thrombophlebitis occurred at the bryostatin 1 infusion site of patients treated at all dose levels up to 50 micrograms m-2, attributable to the 60% ethanol diluent in the bryostatin 1 infusion. Subsequent patients treated at 50 and 65 micrograms m-2 received treatment with an intravenous normal saline flush and they did not develop these complications. Significant decreases of the platelet count and total leucocyte, neutrophil and lymphocyte counts were seen in the first 24 h after treatment at the dose of 65 micrograms m-2. Immediate decreases in haemoglobin of up to 1.9g dl-1 were also noted in patients treated with 65 micrograms m-2, in the absence of clinical evidence of bleeding or haemodynamic compromise. No effect was observed on the incidence of haemopoietic progenitor cells in the marrow. Some patients' neutrophils demonstrated enhanced superoxide radical formation in response to in vitro stimulation with opsonised zymosan (a bacterial polysaccharide) but in the absence of this additional stimulus, no bryostatin 1 effect was observed. Lymphocyte natural killing activity was decreased 2 h after treatment with bryostatin 1, but the effect was not consistently seen 24 h or 7 days later. With the dose schedule examined no antitumour effects were observed. We recommend that bryostatin 1 is used at a dose of 35 to 50 micrograms m-2 two weekly in phase II studies in patients with malignancies including lymphoma, leukaemia, melanoma or hypernephroma, for which pre-clinical investigations suggest antitumour activity.


Br. J. Cancer (1993), 68, 418-424 © Macmillan Press Ltd., 1993

### A phase I study of intravenous bryostatin 1 in patients with advanced cancer

J. Prendiville[1], D. Crowther[1], N. Thatcher[1], P.J. Woll[1], B.W. Fox[2], A. McGown[2], N. Testa[3], P. Stern[4], R. McDermott[4], M. Potter[5] & G.R. Pettit[6]

*CRC Departments of [1]Medical Oncology, [2]Experimental Chemotherapy, [3]Experimental Haematology and [4]Immunology, Paterson Institute and Christie Hospital NHS Trust, Manchester, UK, [5]Department of Immunology, Hope Hospital, Salford, UK and [6]Cancer Research Institute and Department of Chemistry, Arizona State University, Tempe, Arizona, USA.*

**Summary** Bryostatin 1 is a novel antitumour agent derived from *Bugula neritina* of the marine phylum Ectoprocta. Nineteen patients with advanced solid tumours were entered into a phase I study to evaluate the toxicity and biological effects of bryostatin 1. Bryostatin 1 was given as a one hour intravenous infusion at the beginning of each 2 week treatment cycle. A maximum of three treatment cycles were given. Doses were escalated in steps from 5 to 65 $\mu g\ m^{-2}$ in successive patient groups. The maximum tolerated dose was 50 $\mu g\ m^{-2}$. Myalgia was the dose limiting toxicity and was of WHO grade 3 in all three patients treated at 65 $\mu g\ m^{-2}$. Flu-like symptoms were common but were of maximum WHO grade 2. Hypotension, of maximum WHO grade 1, occurred in six patients treated at doses up to and including 20 $\mu g\ m^{-2}$ and may not have been attributable to treatment with bryostatin 1. Cellulitis and thrombophlebitis occurred at the bryostatin 1 infusion site of patients treated at all dose levels up to 50 $\mu g\ m^{-2}$, attributable to the 60% ethanol diluent in the bryostatin 1 infusion. Subsequent patients treated at 50 and 65 $\mu g\ m^{-2}$ received treatment with an intravenous normal saline flush and they did not develop these complications. Significant decreases of the platelet count and total leucocyte, neutrophil and lymphocyte counts were seen in the first 24 h after treatment at the dose of 65 $\mu g\ m^{-2}$. Immediate decreases in haemoglobin of up to 1.9g $dl^{-1}$ were also noted in patients treated with 65 $\mu g\ m^{-2}$, in the absence of clinical evidence of bleeding or haemodynamic compromise. No effect was observed on the incidence of haemopoietic progenitor cells in the marrow. Some patients' neutrophils demonstrated enhanced superoxide radical formation in response to *in vitro* stimulation with opsonised zymosan (a bacterial polysaccharide) but in the absence of this additional stimulus, no bryostatin 1 effect was observed. Lymphocyte natural killing activity was decreased 2 h after treatment with bryostatin 1, but the effect was not consistently seen 24 h or 7 days later. With the dose schedule examined no antitumour effects were observed. We recommend that bryostatin 1 is used at a dose of 35 to 50 $\mu g\ m^{-2}$ two weekly in phase II studies in patients with malignancies including lymphoma, leukaemia, melanoma or hypernephroma, for which pre-clinical investigations suggest antitumour activity.

Bryostatin 1 is a natural product isolated from the marine invertebrate *Bugula neritina*, a member of the phylum Ectoprocta (Pettit *et al.*, 1982). The drug has a complex macrocyclic lactone stucture with a molecular weight of 904 daltons (see Figure 1) and is the prototype of a novel family of potent activators of protein kinase C (PKC: Berkow & Kraft, 1985; Fields *et al.*, 1988). Bryostatin 1, in common with phorbol esters which are also potent activators of PKC (Castagna *et al.*, 1982), elicits a wide range of biological responses including induction of differentiation, haemopoietic stimulation, platelet aggregation and immunoenhancing activity. However in a number of systems bryostatin 1 behaves differently from the phorbol esters. Unlike phorbol esters, bryostatin 1 does not induce differentiation of human bronchial epithelium (Jetten *et al.*, 1989) or primary mouse epidermal cells (Sako *et al.*, 1987) and pre-treatment with bryostatin 1 can serve to inhibit the phorbol ester response. In addition, bryostatin 1 can cause protein phosphorylation at doses that require a far greater molar concentration of phorbol ester to achieve the effect (Fields *et al.*, 1988; Warren *et al.*, 1988). Furthermore, unlike phorbol ester, bryostatin 1 is inactive as a complete tumour promoter or carcinogen and acts to inhibit the tumour promoting properties of phorbol esters (Hennings *et al.*, 1987).

Bryostatin 1 has been shown to have significant *in vitro* antineoplastic effects against murine and human leukaemia cell lines, the L10A B-cell lymphoma, the M5076 reticulum cell sarcoma, ovarian carcinoma, the melanoma cell lines SK-MEL5 and B16, the renal cell line A549 and the lung carcinoma cell line A704 (NCI Antitumour Screening Program; Pettit *et al.*, 1982; Dale & Gescher, 1989; Kraft *et al.*, 1989; Hornung *et al.*, 1992). In addition, significant antitumour activity has been observed in *in vivo* murine models for leukaemia, ovarian carcinoma, B-cell lymphoma, reticulum cell sarcoma and melanoma (Pettit *et al.*, 1982;

**Figure 1** Bryostatin 1 structure.

Correspondence: J. Prendiville, CRC Department of Medical Oncology, Christie Hospital NHS Trust, Wilmslow Road, Manchester M20 9BX, UK.
Received 20 January 1993; and in revised form 6 April 1993.

Schuchter *et al.*, 1991; Hornung *et al.*, 1992). Recently it has been demonstrated in mice that there is a close correlation between the *in vitro* and *in vivo* antitumour effect of bryostatin 1, suggesting a direct mechanism of action for the drug *in vivo* (Hornung *et al.*, 1992).

Numerous studies have demonstrated the potent differentiating effect of bryostatin 1, particularly on fresh human acute and chronic myelogenous and lymphocytic leukaemias (Kraft *et al.*, 1989; Lilly *et al.*, 1990; Gignac *et al.*, 1990; Lilly *et al.*, 1991). However, bryostatin 1 has shown varying ability to differentiate immortal HL 60 chronic myelomonocytic leukaemia cell lines, ranging from some differentiation to little or no effect (Kiss *et al.*, 1987*a*; Kiss *et al.*, 1987*b*; Kraft *et al.*, 1989; Stone *et al.*, 1988; William *et al.*, 1988; Warren *et al.*, 1988). The terminal differentiating effect of bryostatin 1 would appear to produce cytostasis in immortal human cell lines (Jones *et al.*, 1990) but is largely cytotoxic in a variety of fresh human myeloid leukaemia cells (Lilly *et al.*, 1990; Lilly *et al.*, 1991). Here it has been suggested that the differential effect of bryostatin 1 on fresh and immortal leukaemia cells may exist because of differential expression of PKC isoenzymes (Lilly *et al.*, 1991). Bryostatin 1 also induces terminal differentiation of other cells, including conversion of immortal high grade Burkitt lymphoma cells to cells with surface polypeptides characteristic of intermediate grade cells (Al-Katib *et al.*, 1990) and transient transformation of immortal human neuroblastoma cells to mature ganglion cells (Jalava *et al.*, 1990).

As a consequence of the potent antineoplastic and differentiating effects of this novel agent, we have undertaken a phase I study of intravenous bryostatin 1 in patients with advanced cancer.

### Materials and methods

### *Drug supply and formulation*

The bryostatin 1 used in this phase I trial was extracted from *Bugula neritina*. The extraction was undertaken at the NCI-Frederick Cancer Research Facility, Frederick, MD 21701, USA. Formulation and stability studies were carried out by Dr John Slack at the CRC Experimental Cancer Chemotherapy Research Group, Pharmaceutical Sciences Institute, Aston University, Aston Triangle, Birmingham, B4 7ET, UK.

### *Physical properties*

Bryostatin 1 is a colourless, odourless, crystalline substance, relatively insoluble in water and normal saline, but very soluble in 100% ethanol (greater than 4000 $\mu g\ ml^{-1}$). The solubility of bryostatin 1 in a solution of 60% ethanol and 40% normal saline is 97 $\mu g\ ml^{-1}$. Bryostatin 1 is formulated in 1.5 ml of a 0.1 $mg\ ml^{-1}$ solution in 100% ethanol BP in clear glass ampoules (i.e. 0.15 mg per ampoule). A 0.1 mg $ml^{-1}$ solution in absolute ethanol BP shows no sign of degradation after 3 weeks at 50° or 20°C in daylight or after eight months at −20°C. Bryostatin 1 is stable for 24 h in a 60% ethanol solution in 0.9% normal saline in a polypropylene syringe.

### *Preclinical toxicology*

Preclinical toxicology on bryostatin 1 was carried out in both mice and rats by BIBRA, Carshalton, Surrey, UK and sponsored by the Cancer Research Campaign (CRC) UK. Analysis of the murine mortality data using computer linear regression techniques gave an $LD_{10}$ value of 0.029 $mg\ kg^{-1}$ (95% confidence limits of 0.022–0.037 $mg\ kg^{-1}$) and an $LD_{50}$ value of 0.038 $mg\ kg^{-1}$ (95% confidence limits of 0.033–0.042 $mg\ kg^{-1}$). Analysis of the rat mortality data by similar techniques gave an $LD_{10}$ value of 0.045 $mg\ kg^{-1}$ and an $LD_{50}$ value of 0.068 $mg\ kg^{-1}$. Following treatment with bryostatin 1 most of the animals showed initial signs of lethargy, unsteady movement and in some cases haematuria. The majority of deaths occurred within one day of dosing with animals not recovering from the lethargy and unsteady movement seen at the time of dosing. Animals showed a slight decrease in bodyweight at the beginning of the observation period but recovered to control weights by the end of the study. The main effects on organ weights were an increase in liver weight on day 15 and an increase in spleen weight relative to bodyweight on day 29. The haematological data in rats showed the platelet numbers to be significantly reduced on day three of the study with a return to normal levels on day seven. The number of circulatory lymphocytes was also significantly reduced on day three and remained significantly lower in most of the animals throughout the 29 day study period. Rats which died soon after treatment were found to have haemorrhage in the lung, muscle and thymus and perivascular oedema and intravascular fibrous deposition in the lung. Mice were not subjected to haematological investigation and mice dying soon after bryostatin 1 dosing were not subjected to gross necropsy examination. Most animals were found to have significant tail vein necrosis which may have been solely due to the ethanol diluent used in the administration of bryostatin 1.

### *Study design*

Bryostatin 1 was administered as an intravenous infusion of 2 ml of 60% ethanol BP in 40% normal saline over one hour. Patients received a single infusion on day one of each two week treatment cycle. A maximum of three treatment cycles (i.e. three bryostatin 1 infusions) was given. One tenth of the murine $LD_{10}$ value was 5 $\mu g\ m^{-2}$ and this was chosen as the starting dose. The study design was for three patients to be entered at each of the following dose levels: 5, 10, 20, 35, 50 and 65 $\mu g\ m^{-2}$. There was no dose escalation within the same patient. The study endpoint was a maximum tolerated dose (MTD) resulting in a severe or life threatening toxicity (WHO grade 3 or 4) in any system in 66% of patients.

### *Patients*

Nineteen patients were entered into this study. All had advanced solid tumours for which conventional therapy was not available or had failed (see Table I). Inclusion criteria were a Karnofsky performance status of ⩾70, age greater than 18 years, life expectancy of at least three months and a negative stool guaic. The patients had to have normal coagulation (prothrombin time and partial thromboplastin

**Table I** Patient characteristics

| *Pt. No.* | *Diagnosis* | *Bryo Dose* $\mu gm^{-2}$ | *Completion of treatment* |
|---|---|---|---|
| 1 | Ovar Ca. | 5 | Yes |
| 2 | Ovar Ca. | 5 | Yes |
| 3 | Ovar Ca. | 5 | Yes |
| 4 | Ovar Ca. | 10 | Yes |
| 5 | Mesothelioma | 10 | No (Disease progression) |
| 6 | Mesothelioma | 10 | Yes |
| 7 | Mesothelioma | 20 | Yes |
| 8 | Mesothelioma | 20 | Yes |
| 9 | Mesothelioma | 20 | Yes |
| 10 | Ovar Ca. | 35 | Yes |
| 11 | Mesothelioma | 35 | Yes |
| 12 | Ovar Ca. | 50 | Yes |
| 13 | Ovar Ca. | 50 | No (Disease progression) |
| 14 | Ovar Ca. | 50 | Yes |
| 15 | Sarcoma | 50 | Yes |
| 16 | Sarcoma | 50 | No (Disease progression) |
| 17 | Colon Ca. | 65 | Yes |
| 18 | Ovar Ca. | 65 | No (Toxicity) |
| 19 | Ovar Ca. | 65 | No (Toxicity) |

Pt. No = patient trial number; bryo = bryostatin 1; ovar ca = ovarian carcinoma. Reasons for not completing treatment indicated in parenthesis.

time $\leqslant$ 1.2 times the laboratory controls), normal renal function (serum creatinine $\leqslant 0.12$ mmol $l^{-1}$) and adequate hepatic function (bilirubin $\leqslant 25$ μmol $l^{-1}$ and serum liver transaminases $< 2.0$ times normal). Patients were advised not to consume aspirin because of the risk of haemorrhagic gastritis. Patients were excluded from the study if they had received any other antitumour treatment within 3 weeks of study entry, had major surgery within four weeks of study entry, had brain metastases or a known seizure disorder, or were fertile men and women not using an acceptable method of contraception, seropositive for HIV or had other serious intercurrent illness. Any patient with a history of peptic ulceration or gastrointestinal bleeding or a history of any bleeding disorder was also excluded.

*Clinical and laboratory monitoring*

Regular haematological and biochemical investigations were performed before and during treatment with bryostatin 1. These included full blood counts, white cell differentials, measurement of prothrombin and partial thromboplastin times, full biochemistry screen (including liver function tests) and urinalysis. Because of the risk of platelet aggregation, additional full blood counts were performed at ½, 1, 2 and 4 h after each bryostatin 1 infusion. The clinical state of the patients was monitored by physical examinations, recordings of weight, blood pressure, radial pulse and oral temperature. Creatinine phosphokinase levels and electromyography were performed in patients treated at the highest dose level (65 μg $m^{-2}$).

*Clonogenic assays*

Bone marrow aspirates were taken under local anaesthetic from the posterior superior iliac crest for *in vitro* clonogenic assays before treatment and 24 h after the final bryostatin 1 infusion. Twenty ml of heparinised venous blood were collected on the same days. Bone marrow cells for *in vitro* manipulation were collected in Iscove's medium (Gibco) with 50 units of preservative free heparin (CP Pharmaceuticals Ltd). Three aliquots of this suspension were prepared and clonal assays performed as described previously (Testa, 1985) with minor modifications. Briefly, red cells were removed by sedimentation in 0.1% methylcellulose over 30 min at room temperature. The stromal cell population (CFU-F, Colony forming unit-fibroblast) was assayed by suspending cells at $1 \times 10^5$ $ml^{-1}$ in 5 ml of 15% horse serum (Medical Veterinary Supplies) in Iscove's medium in flasks (Falcon T25) gassed with 5% $CO_2$ and incubating for 10 days at 37°C. The flasks were washed with phosphate buffered saline and the adherent cells fixed in methanol and stained with Crystal Violet. Colonies containing more than 50 fibroblasts were scored. The Ficoll (Flow) separated mononuclear fraction of the second aliquot of bone marrow cells was plated at $10^5$ cells $ml^{-1}$ in 1.2% methycellulose, 10% conditioned medium from the 5637 bladder carcinoma cell line (as a source of colony stimulating factors), two units of recombinant erythropoietin (Terry Fox Lab), 1% bovine serum albumin (Sigma) and 30% foetal calf serum (Flow Laboratories) in Iscove's medium. Colonies of more than 50 cells were scored using standard criteria as BFU-E (Burst forming unit–erythroid), GM-CFC (Granulocyte macrophage-colony forming cells) and Mix-CFC (Mixed lineage – colony forming cells) after 14 days of incubation at 37°C in a humidified incubator gassed with 5% $CO_2$ and 5% $O_2$ in nitrogen.

*NK assays*

Venous blood taken pre-treatment and 2 h, 24 h and 7 days following treatment was used to assess the immunological responses to byrostatin 1. Peripheral blood mononuclear cells (PBMC) were isolated from 40 ml of heparinised blood by lymphocyte separation medium (Flow Laboratories).

Fresh PBMC were effectors in a 4 h $^{51}Cr$ release assay to assess the level of NK and endogenous LAK activity against K562, an erythroleukaemia cell line (NK sensitive and LAK sensitive) and Daudi, a Burkitt's lymphoma cell line (NK resistant and LAK sensitive). PBMC were assayed in triplicate at effector to target cell ratios of between 40:1 and 10:1 with $5 \times 10^3$ $^{51}Cr$ labelled target cells per well. These were then incubated at 37°C with 5% $CO_2$ overlay for 4 h. Cytotoxicity was calculated according to the formula:

$$\% \text{ cytotoxicity} = \frac{^{51}\text{Cr release test - spontaneous } ^{51}\text{Cr release}}{\text{maximum } ^{51}\text{Cr release - spontaneous } ^{51}\text{Cr release}}$$

Spontaneous release was obtained by incubating target cells with medium only and maximum release by incubation of target cells with 2% (v/v) Tween 20 (Sigma) in PBS. A more detailed description of this method is given by Ghosh *et al.* (1989).

*Polymorph function tests*

*Cells* Leucocyte populations from peripheral blood were prepared by dextran sedimentation of heparinised blood and residual red cells removed by $H_2O$ lysis. Cells were washed twice in Hanks balanced salt solution (Flow Labs), resuspended in HEPES buffered RPMI-H medium (RPMI 1640, Flow Labs) and counted on a haemocytometer.

*Polymorph phagocytic function* Phagocyte function was measured essentially as described by Easmon *et al.* (1980) by the technique of luminol-dependent chemiluminescence using zymosan as the stimulating agent. The test was performed using disposable polystyrene cuvettes (Clinicon) containing 700 μl luminol (Sigma $10^{-4}$ M in RPMI-H), 200 μl of freshly opsonised zymosan (Zymosan A, Sigma) and 100 μl cell suspension (containing $5 \times 10^6$ polymorphs $ml^{-1}$). Cuvettes were counted repeatedly at 3 min intervals for 30 min using a LKB 1251 Luminometer and the results expressed as mean values for the peak chemiluminescence (in mV). All samples were set up in duplicate.

**Results**

Nineteen patients were entered into this study, three at each of the dose levels 5, 10 and 20 μg $m^{-2}$, two at 35 μg $m^{-2}$, five at 50 μg $m^{-2}$ and three at 65 μg $m^{-2}$ (see Table I). Twelve patients had received prior intensive chemotherapy. Six patients with mesothelioma and one patient with colon cancer had received no prior chemotherapy or radiotherapy.

*Toxicity*

The severity and duration of clinical toxicity was dose related. Several patients developed increasing severity of symptoms with repeated injections of bryostatin 1. Flu-like symptoms of maximum WHO grade 2, (ie moderate severity), such as lethargy, fever, sweats, rigors, rhinitis and headache accounted for most of the clinical toxicity (see Table II). Hypotension, of maximum WHO grade 1, occurred in six patients. The dose limiting toxicity was myalgia, often associated with mild to moderate muscle tenderness. Several patients complained of retro-orbital pain on moving their eyes, and upper dysphagia, both of which we attributed to myalgia in these areas. One patient developed WHO grade 2 myalgia at the starting dose level of 5 μg $m^{-2}$. Other patients treated at 5 μg $m^{-2}$ and patients treated at 10 and 20 μg $m^{-2}$ did not develop this side effect. Both patients treated at the dose level of 35 μg $m^{-2}$ and three of the patients at 50 μg $m^{-2}$ developed myalgia which was of maximum WHO grade 2. All three patients treated at 65 μg $m^{-2}$ developed WHO grade 3 myalgia which necessitated confinement to bed for one or more days, or which required significant amounts of analgesia to relieve the discomfort. One of these three patients developed WHO grade 3 myalgia following the first cycle of treatment and the other two

**Table II** Clinical toxicity

| | *5 μg m⁻² day⁻¹ 9 cycles (3 pts) No. cycles/max. WHO* | | | | *10 μg m⁻² day⁻¹ 7 cycles (3 pts) No cycles/max. WHO* | | | | *20 μg m⁻² day⁻¹ 9 cycles (3 pts) No. cycles/max. WHO* | | | |
|---|---|---|---|---|---|---|---|---|---|---|---|---|
| | *1* | *2* | *3* | *4* | *1* | *2* | *3* | *4* | *1* | *2* | *3* | *4* |
| Lethargy | 1 | 2 | | | | 4 | | | | 1 | | |
| Fever | 3 | | | | 2 | 1 | | | 2 | | | |
| Hypotension | | | | | 3 | | | | 4 | | | |
| Sweats | | | | | | 2 | | | | | | |
| Rigors | | | | | | | | | | | | |
| Rhinitis | | | | | | | | | | | | |
| Headache | | | | | | | | | | | | |
| Myalgia | 1 | | | | | | | | | | | |
| Retro-orbital pain | | | | | | | | | | | | |
| Dysphagia | | | | | | | | | | | | |

| | *35 μg m⁻² day⁻¹ 6 cycles (2 pts) No. cycles/max. WHO* | | | | *50 μg m⁻² day⁻¹ 12 cycles (5 pts) No cycles/max. WHO* | | | | *65 μg m⁻² day⁻¹ 6 cycles (3 pts) No. cycles/max. WHO* | | | |
|---|---|---|---|---|---|---|---|---|---|---|---|---|
| | *1* | *2* | *3* | *4* | *1* | *2* | *3* | *4* | *1* | *2* | *3* | *4* |
| Lethargy | | 5 | | | 1 | | | | 2 | 4 | | |
| Fever | | | | | 3 | 4 | | | | 3 | | |
| Hypotension | | | | | 1 | | | | | | | |
| Sweats | | | | | | 1 | | | 1 | 1 | | |
| Rigors | | | | | | 1 | | | 1 | 1 | | |
| Rhinitis | 2 | | | | | | | | | | | |
| Headache | | | | | 2 | 2 | | | | 3 | | |
| Myalgia | | 4 | | | 1 | 6 | | | | 3 | 3 | |
| Retro-orbital pain | 2 | 2 | | | | | | | | | | |
| Dysphagia | | 1 | | | | | | | | | | |

No. cycles/max. WHO = the maximum toxicity attained during a cycle of treatment and the number of cycles in which this occurred.

patients following the second and third cycles of treatment respectively. They were not given subsequent cycles of treatment. Several patients complained of myalgia getting worse with successive cycles of treatment but this was not true of all patients. The pathogenesis of the myalgia is unknown. Creatinine phosphokinase levels remained within the normal range in the three patients who developed WHO grade 3 myalgia. Electromyography was performed also in all three patients and was normal in two. The third patient's electromyographic trace was slightly abnormal and showed several areas of muscle where there were short polyphasic units and which could be compatable with some patchy myositis.

Cellulitis and thrombophlebitis at the bryostatin 1 infusion site complicated 19 of the total 36 cycles of treatment given to the patients treated up to 35 μg m$^{-2}$ and two of the patients treated at 50 μg m$^{-2}$. These complications occurred at all dose levels but slightly more regularly at the higher dose levels. We believe that the ethanol diluent was the major cause of the phlebitis and cellulitis when it occurred but we cannot exclude bryostatin 1 itself as a contributory factor. Subsequent patients treated at 50 and 65 μg m$^{-2}$ received an intravenous normal saline infusion with their bryostatin 1 treatment in order to flush the bryostatin 1 solute and ethanol diluent from the treatment vein. There was only one case of mild phlebitis out of a total 13 cycles of treatment given in this way.

There were no apparent effects on coagulation, serum biochemistry or liver function assessed regularly following treatment with bryostatin 1.

### *Effects of bryostatin 1 on blood count*

There were no significant changes in any of the haematologic parameters during the administration of bryostatin 1 at doses of 5, 10, 20, 35 and 50 μg m$^{-2}$ day$^{-1}$. The three patients treated at the dose of 65 μg m$^{-2}$ showed decreases of 34–44% in platelet counts with the nadir occurring in the first 4 h. Recovery of the platelet count was usually taking place at 24 h following treatment and full recovery was usually evident at one week (Figure 2).

A reduction in the neutrophil count also occurred in the first 24 h following administration with decreases of 44–64% at 65 μg m$^{-2}$ (Figure 3). Reductions in the lymphocyte count also occurred with decreases of 55–88% at 65 μg m$^{-2}$ (Figure 3).

Of particular interest were immediate decreases in haemoglobin values observed in the first 4 h after treatment with bryostatin 1, most notable in patients treated at the dose of 65 μg m$^{-2}$ with decreases of up to 1.9 gdl$^{-1}$ (see Figure 4). Unlike decreases in platelet, neutrophil and lymphocyte counts, recovery of the haemoglobin values was not noted in the next 2 weeks. There was no clinical evidence of bleeding and the patients showed no evidence of haemodynamic compromise.

### *Bone marrow clonogenic assays*

*In vitro* clonogenic assays of haemopoietic progenitors were performed on bone marrow aspirates taken before the first bryostatin 1 infusion and 24 h after the final treatment from

**Figure 2** Platelet counts measured over the first 24 h and then at one week following administration of the first cycle of bryostatin 1 in the three patients treated at the dose of 65 μg m$^{-2}$.

Figure 3 Neutrophil (———) and lymphocyte (------) counts measured over the first 24 h and then at one week following administration of the first cycle of bryostatin 1 to the three patients treated at the dose of 65 $\mu$g m$^{-2}$.

Figure 4 Haemoglobin measurements over the first 24 h and then at one week following administration of the first cycle of bryostatin 1 to the three patients treated at the dose of 65 $\mu$g m$^{-2}$.

one patient treated at the dose of 50 $\mu$g m$^{-2}$ and two patients treated at 65 $\mu$g m$^{-2}$. No *in vivo* effect of bryostatin 1 on haemopoietic progenitor cells was demonstrated (data not shown).

### *NK activity*

In patients 14 to 18, a decrease in the NK activity (against K562) was observed 2 h following treatment (see Table IV). In all cases, subsequent levels of NK were low but gradually increased over the 7 day observation period. Endogenous LAK activity (against Daudi cells) followed a similar trend. No dose-dependent differences were observed in NK activity.

### *Neutrophil chemiluminescence*

Maximum neutrophil superoxide radical formation detected by chemiluminescence was measured pre treatment and at 2 h and 24 h following a single bryostatin 1 infusion in patients treated at 20, 35 50 and 65 $\mu$g m$^{-2}$. No significant effect of bryostatin 1 was observed on neutrophils in the absence of opsonised zymosan (a bacterial polysaccharide). If neutrophils were additionally stimulated, with opsonised zymosan significant increases in the maximum neutrophil chemiluminescence recorded were observed in some patients (see Table III). Increases or decreases of greater than 25% are thought to be within the error range of the assay and are not shown.

**Table III** Percentage increase from baseline value of maximum neutrophil superoxide radical formation (as detected by chemiluminescence) following *in vitro* stimulation with opsonised zymosan

| Pt No | Byro $\mu$g m$^{-2}$ | *Percentage charge over pre treatment level* 2 hours post treatment | 24 hours post treatment |
|---|---|---|---|
| 7 | 20 | +29 | +46 |
| 8 | 20 | N.C. | N.C. |
| 10 | 35 | N.C. | N.C. |
| 11 | 35 | +26 | +33.2 |
| 12 | 50 | +181 | +113 |
| 13 | 50 | N.C. | N.C. |
| 14 | 50 | N.C. | N.C. |
| 15 | 50 | N.C. | +59 |
| 16 | 50 | +30 | +45.2 |
| 17 | 65 | N.C. | N.C. |
| 18 | 65 | N.C. | +33.4 |
| 19 | 65 | N.C. | +40.2 |

Pt No = patient trial number, bryo 1 = bryostatin 1; N.C. = no change from pre treatment result and includes increases or decreases of up to 25%.

### Discussion

Bryostatin 1 is a potent activator of PKC which elicits a wide range of biological effects, including platelet aggregation, enhancement of the production and function of haemopoietic growth factors, activation of intact polymorphonuclear leucocytes, pleiotropic immunoenhancing effects on both T and B lymphocytes, induction of differentiation and potent *in vitro* and murine *in vivo* antineoplastic effects. *In vitro* studies have shown bryostatin 1 to be extremely active in the nanomolar range. Preclinical toxicology on bryostatin 1 confirmed similar *in vivo* potency with a murine $LD_{10}$ value of 0.029 mg kg$^{-1}$. Bryostatin 1 therefore entered phase 1 clinical trial at a dose of 5 $\mu$g m$^{-2}$.

Flu-like symptoms such as lethargy, fever, sweats, rigors, rhinitis and headache accounted for the majority of clinical toxicity but they were of maximum WHO grade 2. Mild hypotension occurred in six patients treated at doses up to and including 20 $\mu$g m$^{-2}$ and may not have been attributable to treatment with bryostatin 1. Myalgia, aggravated by exercise, emerged as the dose limiting toxicity usually occurring about 48 h after threatment and lasting up to several weeks at the highest dose levels. Several patients complained of increasing myalgia with successive cycles of treatment. The pathogenesis of this toxicity is uncertain and there was no clear evidence compatible with either myositis or myolysis. Muscle tenderness was evident in many patients but was not a prominent feature. Electromyography (EMG) demonstrated some evidence of patchy myositis in one of three patients treated at 65 $\mu$g m$^{-2}$. Serum creatinine phosphokinase (CPK) levels remained within the normal range in the same three patients. *In vitro* work has shown that bryostatin 1, like other protein kinase C activators, induces electrical membrane instability in genetically normal muscle, leading to an unscheduled series of action potentials (Brinkmeier & Jockusch, 1987). These findings were caused by a drastically lowered sarcolemma chloride conductance. If this was the cause of the myalgia in our patients, one would have expected florid changes on the EMG's which did not occur. Another possible explanation for the myalgia is ischaemia cause by platelet microemboli (i.e. platelet aggregates) but there was no rise in CPK levels secondary to ischaemia-induced necrosis.

Cellulitis and thrombophlebitis at the bryostatin 1 infusion site complicated 53% of cycles of treatment administered without an accompanying normal saline venous flush. We believe that these complications resulted from the 60% ethanol diluent in the bryostatin 1 infusion, though we cannot exclude the possibility that the bryostatin 1 itself may have been a contributing factor.

Bryostatin 7 has been shown to be a potent inducer of human platelet aggregation *in vitro* (Tallant et al., 1987) and we have demonstrated similar activity by bryostatin 1 *in vitro*

**Table IV** Lymphocyte natural killing activity as measured by % cytotoxicity of either K562 or Daudi cells

| Pt No | Byro 1 $\mu g\,m^{-2}$ | Pre treatment K562 | Pre treatment Daudi | Percentage cytotoxicity 2 hours K562 | 2 hours Daudi | 24 hours K562 | 24 hours Daudi | 7 days K562 | 7 days Daudi |
|---|---|---|---|---|---|---|---|---|---|
| 14 | 50 | 4.1 | 2.2 | 0.8 | 4.0 | 12.7 | 1.7 | 5.4 | 1.6 |
| 15 | 50 | 4.8 | 3.0 | 0.0 | 0.0 | 4.2 | 0.1 | 3.0 | 13.3 |
| 16 | 50 | 6.3 | 21.5 | 1.2 | 2.3 | 13.4 | 12.3 | 16.1 | 10.7 |
| 17 | 65 | 21.5 | 35.7 | 7.0 | 5.7 | 11.8 | 5.2 | 7.5 | 5.2 |
| 18 | 65 | 20.3 | 5.8 | 2.5 | 0.0 | 11.8 | 0.11 | 16.1 | 5.3 |
| 19 | 65 | 6.0 | 10.0 | ND | ND | 7.4 | 2.2 | 16.4 | 7.6 |

Pt No = patient trial number; bryo 1 = broystatin 1; ND = no data.

and *in vivo* (data not shown). Mouse and rat toxicity studies have also shown a profound *in vivo* effect of bryostatin 1 on platelets. In this phase 1 study we observed a drop in platelet counts only at the bryostatin 1 dose of 65 $\mu g\,m^{-2}$, occurring usually up to 4 h after treatment. Platelet aggregation was not profound and decreases of only 34–44% in the platelet count were observed. Pre-clinical toxicity studies demonstrated more profound platelet count drops and occasionally with significant internal bleeding, but at relatively higher bryostatin 1 doses than we used in this phase 1 trial. The emergence of dose limiting myalgia has meant that we did not approach bryostatin 1 doses in this trial where we might encounter profound platelet aggregation and run a significant risk of bleeding.

Decreases in the haemoglobin values of up to 1.9 $gdl^{-1}$ in patients treated at the bryostatin 1 dose of 65 $\mu g\,m^{-2}$ are unexplained. There was no clinical evidence of bleeding or haemodynamic compromise. Haemolysis may explain the finding.

Transient reductions in the neutrophil and lymphocyte counts also occurred at the dose of 65 $\mu g\,m^{-2}$ in the first 24 h after treatment. These may have been due to the sequestration of functionally active leucocytes. No evidence of toxicity on haemopoietic progenitor cells was found in clonogenic studies on bone marrow taken from these patients. Preclinical toxicity studies have demonstrated significant reductions in lymphocyte counts lasting up to 29 days after treatment with bryostatin 1, but at relatively higher doses than we achieved in this phase 1 trial.

Bryostatin 1 functionally activates intact human polymorphonuclear leucocytes, enhancing cytotoxicity and release of superoxide radicals and specific granules (May *et al.*, 1987; Berkow & Kraft, 1985). We were unable in this phase I trial to clearly demonstrate enhanced production of superoxide radicals by neutrophils taken from patients treated with bryostatin 1 at doses of 20, 35, 50 and 65 $\mu g\,m^{-2}$. However, if these neutrophils were additionally stimulated *in vitro*, using opsonised zymosan an enhanced production of superoxide radicals were seen in seven patients at doses of 20, 35, 50 and 65 $\mu g\,m^{-2}$. The doses of bryostatin 1 administered in this phase 1 trial were not on their own, enough to stimulate directly the release of superoxide radicals but were sufficient to 'prime' the neutrophils for an enhanced response to a second stimulus.

Bryostatin 1 exerts pleiotropic effects on human lymphocytes *in vitro* including the activation and induction of proliferation of both T and B cells (Trenn *et al.*, 1988; Hess *et al.*, 1988; Drexler *et al.*, 1990) and the induction of interleukin-2 receptors (IL-2R) on T cells (Hess *et al.*, 1988). Conflicting evidence exists on the ability of bryostatin 1 to enhance or trigger human lymphocyte mediated cytotoxicity. Trenn *et al.*, 1988, have reported on the ability of bryostatin 1 to trigger cytotoxic T lymphocyte (CTL) development in naive resting lymph node cells and to trigger cytotoxicity of CTL clones against antigen (Ag) – non bearing target cells. However, CTL cytotoxicity against Ag-specific target cells was inhibited. Bryostatin 1 was also shown to greatly enhance the efficiency of recombinant interleukin-2 (rIL-2) in triggering development of *in vivo* primed CTL during *in vitro* incubation. In all patients monitored in our study the NK activity decreased initially following administration of bryostatin 1. However, since the actual values obtained for % cytotoxicity were low the significance of these differences has to be interpreted with caution. Similar observations were made *in vitro* by Tilden and Kraft (1991) who found a suppression of NK activity following an 18 h preincubation of lymphocytes with $10^{-8}$ M bryostatin 1. These authors also observed reduced cytotoxic activity of LAK effector cells following coincubation with rIL-2 and $10^{-8}$ M bryostatin 1. It will be of interest to study the *in vitro* LAK activities of lymphocyte populations of patients receiving bryostatin 1.

Bryostatin 1 exerts a wide variety of stimulatory effects on bone marrow progenitor cells and peripheral blood cells. Originally bryostatin 1 was reported to enhance proliferation of human and murine haemopoietic progenitor cells, including committed myeloid progenitor cells (CFU-GM) and early and late erythroid progenitors (BFU-E and CFU-E: May *et al.*, 1987; Leonard *et al.*, 1988; Gebbia *et al.*, 1988). Subsequently it has been shown that this is an indirect effect and includes the augmentation of the response by haemopoietic progenitors to the haemopoietic growth factors, granulocyte macrophage colony stimulation factor (GM-CSF) and interleukin-3 (IL-3: McCrady *et al.*, 1991; Sharkis *et al.*, 1990) and perhaps also the stimulation of accessory cell populations, including T cells, to produce GM-CSF and IL-3 (Sharkis *et al.*, 1990; Leonard *et al.*, 1988). We were unable in this phase 1 trial to demonstrate changes in the incidence of progenitor cells in the marrow.

Pre-clinical studies have demonstrated that bryostatin 1 is an extremely potent antineoplastic agent. The marine animal origin, macrocylcic lactone structure and PKC receptor mechanism make it one of the most unusual anti-tumour agents to become available for phase I clinical testing. In addition, only extremely low doses are required to produce a biological effect *in vivo*. We recommend phase II clinical testing at a bryostatin 1 dose of 35 to 50 $\mu g\,m^{-2}$ two weekly in patients with malignancies including lymphoma, leukaemia, melanoma or hypernephroma for which preclinical studies suggest antitumour activity. We are currently evaluating weekly treatments by 24 h infusion at a bryostatin 1 dose of 25 to 50 $\mu g\,m^{-2}$ in patients with malignancies including lymphoma.

The authors thank A. Campbell for technical assistance and Research Nursing Sisters V. Goode, B. Traynor and A. Watson. D. Crowther, N. Thatcher, P. Woll, B. Fox, A. McGown, N. Testa, P. Stern and R. McDermott are supported by the Cancer Research Campaign.

### References

AL-KATIB, A., MOHAMMAD, R.M., MOHAMED, A.N., PETTIT, G.R. & SENSENBRENNER, L.L. (1990). Conversion of high grade lymphoma tumour cell line to intermediate grade with TPA and bryostatin 1 as determined by polypeptide analysis of 2D gel electrophoresis. *Haemat. Oncol.*, **8,** 81–89.

BERKOW, R.L. & KRAFT, A.S. (1985). Bryostatin, a non-phorbol macrocyclic lactone, activates intact human polymorphonuclear leukocytes and binds to the phorbol ester receptor. *Biochem. Biophys. Res. Commun.*, **131,** 1109–1116.

BRINKMEIR, H. & JOCKUSCH, H. (1987). Activatoris of protein kinase C induce myotonia by lowering chloride conductance in muscle. *Biochem. Biophys. Res. Commun.*, **148**, 1383–1389.

CASTAGNA, M., TAKAI, Y., KAIBUCHI, K., SANO, K., KIKKAWA, U. & NISHIZUKA, Y. (1982). Direct activation of calcium-activated, phospholipid-dependent protein kinase by tumour-promoting phorbol esters. *J. Biol. Chem.*, **257**, 7847–7851.

DALE, I.L. & GESCHER, A. (1989). Effects of activators of protein kinase C, including bryostatins 1 and 2 on the growth of A549 human lung carcinoma cells. *Int. J. Cancer*, **43**, 158–163.

DREXLER, H.G., GIGNAC, S.M., PETTIT, G.R. & HOFFBRAND, A.V. (1990). Synergistic action of calcium ionophore A23187 and protein kinase C activator bryostatin 1 on human B cell activation and proliferation. *Eur. J. Immunol.*, **20**, 119–127.

EASMON, C.S.F., COLE, P.J., WILLIAMS, A.J. & HASTINGS, M. (1980). The measurement of opsonic and phagocytic function by luminol-dependent chemiluminescence. *Immunology*, **41**, 67–74.

FIELDS, A.P., PETTIT, G.R. & MAY, W.S. (1988). Phosphorylation of laminin B at the nuclear membrane by activated protein kinase C. *J. Biol. Chem.*, **263**, 8253–8260.

GEBBIA, V., DI MARCO, P., MICELI, S. REYES, M.B., TERESI, M., BONACCORSO, R., CITARRELLA, P. & RAUSA, L. (1988). The *in vitro* effect of the antineoplastic agent bryostatin 4 on human haematopoietic progenitor cells. *Haematologica*, **73**, 387–391.

GHOSH, A.K., DAZZI, H., THATCHER, N. & MOORE, M. (1989). Lack of correlation between peripheral blood lymphokine activated killer (LAK) cell function and clinical response in patients with advanced malignant melanoma receiving recombinant interluekin 2. *Int. J. Cancer*, **43**, 410–414.

GIGNAC, S.M. BUSCHLE, M., ROBERTS, R.M., PETTIT, G.R., HOFFBRAND, A.V. & DREXLER, H.G. (1990). Differential expression of TRAP isoenzyme in B-CLL cells treated with different inducers. *Leuk. Lymphoma*, **3**, 19–29.

HENNINGS, H., BLUMBERG, P.M., PETTIT, G.R., HERALD, C.L., SHORES, R. & YUSPA, S.H. (1987). Bryostatin 1, an activator of protein kinase C, inhibits tumour promotion by phorbol esters in SENCAR mouse skin. *Carcinogenesis*, **8**, 1343–1346.

HESS, A.D., SILANSKIS, M.K., ESA, A.H., PETTIT, G.R. & MAY, W.S. (1988). Activation of human T lymphocytes by bryostatin. *J. Immunology*, **141**, 3263–3269.

HORNUNG, R.L., PEARSON, J.W., BECKWITH, M. & LONGO, D.L. (1992). Preclinical evaluation of bryostatin as an anticancer agent against several murine tumour cell lines: *in vitro* versus *in vivo* activity. *Cancer Res.*, **52**, 101–107.

JALAVA, A.M., HEIKKILA, J., AKERLIND, G., PETTIT, G.R. & AKERMAN, K.E.D. (1990). Effects of bryostatins 1 and 2 on morphological and functional differentiation of SH-SY5Y human neuroblastoma cells. *Cancer Res.*, **50**, 3422–3428.

JETTEN, A.M., GEORGE, M.A, PETTIT, G.R. & REARICK, J.I. (1989). Effects of bryostatins and retinoic acid on phorbol ester – and diacylglycerol-induced squamous differentiation in human tracheobronchial epithelial cells. *Cancer Res.*, **49**, 3990–3995.

JONES, R.A., DREXLER, H.G., GIGNAC, S.M., CHILD, J.A. & SCOTT, C.S. (1990). *In vitro* beta 2-microglobulin (β2m) secretion by normal and luekaemic B-cells: effects of recombinant cytokines and evidence for a differential response to the combined stimulus of phorbol ester and calcium ionophore. *Br. J. Cancer*, **61**, 675–680.

KISS, Z., DELI, E., SHOJI, M., KOEFFLER, H.P., PETTIT, G.R. & VOGLER, W.R. (1987*a*). Differential effects of various protein C activators on protein phosphorylation in human acute myeloblastic leukaemia cell line KG-1 and its phorbol ester-resistant subline KG-1a. *Cancer Res.*, **47**, 1302–1307.

KISS, Z., DELI, E., GIRARD, P.R., PETTIT, G.R. & KUO, J.F. (1987*b*) Comparative effects of polymyxin B, phorbol ester and bryostatin on protein phosphorylation, protein kinase C translocation, phospholipid metabolism and differentiation of HL60 cells. *Biochem. Biophys. Res. Commun.*, **146**, 208–215.

KRAFT, A.S., WILLIAM, F., PETTIT, G.R. & LILLY, M.B. (1989). Varied differentiation responses of human leukaemias to bryostatin 1. *Cancer Res.*, **49**, 1287–1293.

LEONARD, J.P., MAY, W.S., IHLE, J.N., PETTIT, G.R. & SHARKIS, S.J. (1988). Regulation of haematopoiesis IV: the role of interluekin-3 and bryostatin 1 in the growth of erythropoietic progenitors from normal and anaemic W/W$^v$ mice. *Blood*, **72**, 1492–1496.

LILLY, M., TOMPKINS, C., BROWN, C., PETTIT, G.R. & KRAFT, A. (1990). Differentiation and growth modulation of chronic myelogenous leukaemia cells by bryostatin. *Cancer Res.*, **50**, 5520–5525.

LILLY, M., BROWN, C., PETTIT, G.R. & KRAFT, A. (1991). Bryostatin 1: a potential anti-leukaemic agent for chronic myelomonocytic leukaemia. *Leukemia*, **5**, 283–287.

MAY, W.S., SHARKIS, S.J., ESA, A.H., GEBBIA, M.U., KRAFT, A.S., PETTIT, G.R. & SENSENBRENNER, L. (1987). Antineoplastic bryostatins are multipotential stimulators of human haematopoietic progenitor cells. *Proc. Natl Acad. Sci.* USA, **84**, 8483–8487.

MCCRADY, C.W., STANISWALIS, J., PETTIT, G.R., HOWE, C. & GRANT, S. (1991). Effect of pharmacologic manipulation of protein kinase C by phorbol dibutyrate and bryostatin 1 on the clonogenic response of human granulocyte macrophage progenitors to recombinant GM-CSF. *Br. J. Haematol.*, **77**, 5–15.

PETTIT, G.R., HERALD, C.L., DOUBEK, D.L. & HERALD, D.L. (1982). Isolation and structure of bryostatin 1. *J. Am. Chem. Soc.*, **104**, 6846–6848.

SAKO, T., YUSPA, S.H., HERALD, C.L., PETTIT, G.R. & BLUMBERG, P.M. (1987). Partial parellelism and partial blockade by bryostatin 1 of effects of phorbol ester tumour promoters on primary mouse epidermal cells. *Cancer Res.*, **47**, 5445–5450.

SCHUCHTER, L.M., ESA, A.H., MAY, W., LAULIS, M.K., PETTIT, G.R. & HESS, A.D. (1991). Successful treatment of murine melanoma with bryostatin 1. *Cancer Res.*, **51**, 682–687.

SHARKIS, S.J., JONES, R.J., BELLIS, M.L., DEMETRI, G.D., GRIFFIN, J.D., CIVIN, C. & MAY, W.S. (1990). The action of bryostatin on normal human haematopoietic progenitors is mediated by accessory cell release of growth factors. *Blood*, **76**, 716–720.

STONE, R.M., SARIBAN, E., PETTIT, G.R. & KUFE, D.W. (1988). Bryostatin 1 activates protein kinase C and induces monocyte differentiation of HL-60 cells. *Blood*, **72**, 208–213.

TALLANT, E.A, SMITH, J.B. & WALLACE, R.W. (1987). Bryostatins mimic the effects of phrobol esters in intact human platelets. *Biochem. Biophys. Acta*, **929**, 40–46.

TESTA, N.G. (1985). Clonal assays for haemopoietic and lymphoid cells *in vitro*. In: *Cell Clones: Manual of Mammalian Cell Techniques*. Potten, C.S. & Henry, J.H. (eds). p. 27–43. Churchill Livingstone: New York.

TILDEN, A.B. & KRAFT, A.S. (1991). The effect of bryostatin 1 on human lymphocyte-mediated cytotoxicity. *J. Immunother.*, **10**, 96–104.

TRENN, G., PETTIT, G.R., TAKAYAMA, H., HU-LI, J. & SITKOUSKY, M.U. (1988). Immunomodulating properties of a novel series of protein kinase C activators: the bryostatins. *J. Immunol.*, **140**, 433–439.

WARREN, B.S., KAMANO, Y., PETTIT, G.R. & BLUMBERG, P.M. (1988). Mimicry of bryostatin 1 induced phosphorylation patterns in HL-60 cells by high phorbol ester concentrations. *Cancer Res.*, **48**, 5984–5988.

WILLIAM, F., MROCZKOWSKI, B., COHEN, S. & KRAFT, A.S. (1988). Differentiation of HL-60 cells is associated with an increase in the 35-kDa protein lipocortin 1. *J. Cell. Physiol.*, **137**, 402–410.